# Adaptive Floor Cleaning Strategy by Human Density Surveillance Mapping with a Reconfigurable Multi-Purpose Service Robot

**DOI:** 10.3390/s21092965

**Published:** 2021-04-23

**Authors:** Vinu Sivanantham, Anh Vu Le, Yuyao Shi, Mohan Rajesh Elara, Bing J. Sheu

**Affiliations:** 1 ROAR Lab, Engineering Product Development, Singapore University of Technology and Design, Singapore 487372, Singapore; sivanantham_vinu@mymail.sutd.edu.sg (V.S.); yuyao_shi@sutd.edu.sg (Y.S.); rajeshelara@sutd.edu.sg (M.R.E.); 2 Optoelectronics Research Group, Faculty of Electrical and Electronics Engineering, Ton Duc Thang University, Ho Chi Minh City 700000, Vietnam; 3 Department of Electronics Engineering, Chang Gung University, Taoyuan City 33302, Taiwan; bsheu@mail.cgu.edu.tw

**Keywords:** adaptive multi-robot cleaning startegy, safe social distance surveillance, COVID-19, coverage path planning, reconfigurable robotics

## Abstract

Professional cleaning and safe social distance monitoring are often considered as demanding, time-consuming, repetitive, and labor-intensive tasks with the risk of getting exposed to the virus. Safe social distance monitoring and cleaning are emerging problems solved through robotics solutions. This research aims to develop a safe social distance surveillance system on an intra-reconfigurable robot with a multi-robot cleaning system for large population environments, like office buildings, hospitals, or shopping malls. We propose an adaptive multi-robot cleaning strategy based on zig-zag-based coverage path planning that works in synergy with the human interaction heat map generated by safe social distance monitoring systems. We further validate the proposed adaptive velocity model’s efficiency for the multi-robot cleaning systems regarding time consumption and energy saved. The proposed method using sigmoid-based non-linear function has shown superior performance with 14.1 percent faster and energy consumption of 11.8 percent less than conventional cleaning methods.

## 1. Introduction

The rampant COVID-19 has brought global crisis worldwide by affecting almost all the continents, infecting more than 103 million people and 2.2 million death reports as of 31 January 2021. With the ongoing COVID-19 pandemic situation, following safe social distancing norms, frequent cleaning, and sterilization of the environment has become an indispensable safety measure to mitigate the spread of the virus. The current pandemic situation has already shown numerous instances of how service robots are being used to help and control the virus’s spread [1]. According to TechNavio’s Global Service Robot Market report, the service robotics industry is poised to grow at a compounded rate of 16.5% [2]. One of the significant and vital sectors with an increasing demand for service robots, especially with the ongoing COVID-19 pandemic, is the healthcare industry. In places, like hospitals, with patients entering and leaving, healthcare staff and other co-workers are more exposed to the risk of virus infections. Hence, frequent cleaning and sanitation of the environment are vital for controlling the spread of infections.

Over the past few decades, numerous researches have been done in the development of service robots. Robots have been applied in the domestic and industrial workspaces, such as collaborative robots robotic painting, robotic welding, material removal, part transfer, and machine tending. Recently, the robots, such as AGV and drones, have made great strides in fighting the COVID-19 pandemic [3]. these robots integrated with AI-assisted technology distribute the goods to COVID-19 patients, implements effectively the safe entry check-in body temperature measurement and social distancing, routine sanitize the infected area, frequent cleaning of high touchpoints, like hospital walls, floors, and door handles [4,5,6].

In cleaning applications, area coverage is seen as one of the emerging problems, and exploration for coverage path planning has been studied in depth under various aspects: design, control, autonomy, perception techniques, and multi-robot strategies. For instance, in the design aspects of cleaning robots, Wu et al., in Reference [7], presented the use of vacuum suction and propeller-thrust techniques as adhesion mechanisms for cleaning the vertical surfaces. These adhesion techniques have greater flexibility in controlling the adhesion force exerted on the surface by varying the power supply. Leveraging on the idea of self reconfigurable design mechanisms have been widely explored in-floor cleaning [8], ship hull cleaning maintenance [9,10], staircase cleaning [11], and pavement cleaning [12]. hTetro robot in Reference [8] is a vacuum cleaning autonomous robot that can reconfigure its morphology to access narrow regions that cannot be reached by standard fixed-shaped vacuum cleaning robots. It can achieve a higher cleaning area than a standard automatic vacuum cleaner in the market today. In another work, Vega et al. [13] presented the design and modeling of a self-reconfigurable window cleaning robot named Mantis. Using impellers to hold on to the vertical glass surface, they have demonstrated Mantis to transition from one window panel to another by avoiding the window frame. In Reference [14], Le et al. presented the design and development of a novel self-reconfigurable staircase cleaning robot sTetro. sTetro uses a vertical conveyor belt mechanism to achieve staircase climbing.

To overcome speed constraints and control complexity, various types of locomotion strategies have been proposed. For instance, Gao et al. [15] presented a floor cleaning robot equipped with Swedish wheels to enable Omnidirectional locomotion. The kinematics and the robot’s motion control have been tested to demonstrate locomotion in eight directions without changing the robot’s posture. Miyake et al. [16] analyzed and illustrated the adsorption technique and locomotion mechanism of a window cleaner robot. They utilized a suction cup to adsorb over vertical surfaces, and the two motors fixed in the locomotion module help achieve locomotion over any axis along the plane. Megalingam et al., in Reference [17], presented a staircase cleaning robot equipped with a tracked belt for locomotion and a cleaning brush attached to the front side of the robot supports in climbing up of staircase. In the aspect of autonomy in area coverage-based robots, Kim and Ryan presented a navigation algorithm based on visual SLAM to achieve efficient area coverage for the robots [18]. In Reference [19], Liu et al. introduced a novel coverage path planning strategy for an autonomous cleaner, where they integrated local coverage path planning and random path planning to achieve robustness and efficiency in coverage performance to work in family environments. Implementing autonomous navigation for reconfigurable cleaning robots has been an interesting topic in recent times. Le et al. [20] demonstrated an A-star-based zig-zag global path planning approach for a Tetris-inspired self-reconfigurable cleaning robot (hTetro). They further validated the presented approach to cover narrow spaces leveraging the robot’s shape-shifting capabilities. In other works, Le et al. [6] and Verra et al. [21] presented the idea of adapting tiling theory principles as a coverage path planning strategy for autonomous navigation in Tetris inspired self-reconfigurable robot hTetro. This robot platform is also applied in shortest path planning [5]. They further validated the proposed approach with a set of experimental trials by varying obstacle density in the testbed environment and extended the platform morphologies to hexagon- [22], diamond- [23], and rhombus [6]-based shapes. Regarding the optimal control for tilling robot, Yuyao et al. [24] presented the idea of path tracking control of self-reconfigurable robot hTetro with four differential drive units that address the synchronization issues of linked tiling multi-blocks platforms.

Another critical aspect of cleaning robots is to understand the surroundings and build synergy with their surroundings. So, perception technique is an important aspect that determines the robustness and overall efficiency of the robot. Maryam et al. [25] proposed a CNN-based deep learning approach for a glass facade cleaning robot to detect cracks in glass pane to avoid navigation on the cracked glass. The proposed trained CNN models validated its performance with an accuracy of 90 percent in detecting cracks, while Seul et al. presented a surface texture classification method for autonomous cleaning robots using the Gray Level Co-occurrence Matrix. The proposed approach aids in optimizing cleaning performance for different floor surfaces [26]. In References [27,28], the authors proposed a vision-based Deep Learning (DL) framework to classify, detect and segment the wall/floor surface to perform wall cleaning. The proposed framework is evaluated on a Toyota Human Support Robot. Veera et al. [29] and Muthugala et al. [30] developed the self-evaluating system to validate the cleaning efficiency using corrosion classification deep learning-based model and fuzzy logic model.

While AI and perception techniques are popular in many domains and are an essential aspect to improve the efficiency of the cleaning system, adapting to a multi-robot system is another critical aspect in cleaning robots to improve productivity with fast cleaning approach. Luo et al. [31] proposed a real-time cooperative sweeping strategy of complete coverage path planning for multiple cleaning robots. This approach produces an efficient path that allows robots to avoid collisions with obstacles and other robots and work cooperatively, using biologically inspired neural networks. The approach focuses on improving the efficiency of cleaning with multiple cleaning robots. Using swarm robots by Megalingam et al. [32] is another approach in multi-robot systems to cover a large area in less time by using multiple slave bots controlled by a master bot. In another work, Kurazume et al. [33] developed a cleaning robot system with a cooperative positioning system (CPS). The proposed system focuses on reducing localization errors by using landmarks information from multiple robots in the CPS.

In the COVID-19 pandemic situation, other than frequent cleaning and sanitation of the environment, following safe social distancing measures is also crucial to slow down the virus’s spread. According to the Centre for disease control (CDC) for social distancing measures, a physical distance of at least 6ft is necessary to be maintained between each individual [34]. A survey study in Reference [35] indicated that adopting social distancing measures in Spain has shown significant reductions in daily COVID-19 cases. To ensure safe social distancing measures are appropriately followed, several practices are implemented globally to avoid crowd gathering. For example, employing special officers in public places to monitor the social distancing norms, limiting the maximum number of people allowed in workplaces to restrict crowd gathering, installing safe distance signboards in potentially crowded places [36,37]. However, monitoring and tracking safe social distancing in potentially crowded places, such as hospitals, workplaces, shopping malls, public transport stations, and schools, are quite challenging. Moreover, monitoring safe distance measures is a manual process that can bring the surveillance staff in close proximity with people affected by COVID-19. With the current COVID-19 pandemic, safe social distance monitoring is seen as an emerging problem that employs robotics solutions. Exploring human safe distancing surveillance can be studied under various aspects, including human personal spatial zones, communication frameworks, and perception techniques. In the case of safe social distance surveillance systems, one significant aspect that has been widely studied is the idea of personal human space in the field of cognitive science. Based on human interactions and interpersonal distances maintained between humans Hall et al., in Reference [38], classified personal spatial zone into three categories: public interaction, social interaction, and intimate interaction.

For instance, Reference [39] presented the idea of using personal human space to model social behavior in robots. The proposed model uses a personal space zone between humans to determine the gap between the robot and humans while standing in a line. In another research work, Reference [40] explores the idea of transferring human personal spatial zones to human-robot interactions. A critical aspect of social distance surveillance is communication systems in cellular devices and wearable systems to track humans and control gathering. In Reference [41], the use of UWB in lightweight wearable devices to measure people’s distance is presented. It alerts the people if any person breaks the safe distancing norms. Many countries have been taking the help of technology-based solutions, such as wifi, cellular, and GNSS positioning (localization) systems, to monitor and alert the social distance in public and crowded areas [42,43], while the exploration on the use of drones and IoT-based technologies are gaining popularity for their applications in monitoring crowd control and alert safe distancing between people [37,44]. Adopting AI and other perception-assisted techniques to detect and track human interactions is one viable approach to better perform safe social distance monitoring in dynamic and indoor environments. For example, in Reference [45], a multi-sensor-based human tracking algorithm is proposed. The proposed approach uses two detection layers where a laser range scanner is used to detect human legs and a monocular camera to detect a human face. Further, they fuse the data using an Unscented Kalman filter for human tracking.

Given a large number of applications in the domain of cleaning and safe social distancing surveillance, existing robotic innovations and technologies provide a wide range of solutions to improve productivity and quality. Even though the works mentioned above discuss various aspects of safe distancing surveillance and cleaning robots to improve productivity and quality, they still are primarily limited with performance constraints due to their application-specific design and their capability to handle one specific application. This militates to the use of the different robots for each application. The major hurdles in using individual robots to perform these tasks are that each robot requires expensive and multiple sensors, communication frameworks, navigation hardware, and high-end processors installed throughout the facility. To this end, this work mainly focuses on the development of an intra-reconfigurable robot WASP with a virtually connected multi-robot cleaning system. In this work, we present an adaptive cleaning method for the virtually connected multi-robot cleaning system. The cleaning system works in synergy with the WASP platform’s surveillance module. The surveillance system’s role is to monitor and identify the contaminated regions based on human interaction levels and generate a zig-zag-based coverage path along with motion commands based on adaptive velocity behavior models. Thus, the multi-robot systems proposed in this approach do not require high computational resources and high-cost intuitive sensors for perception. The proposed adaptive cleaning methodology algorithm is simulated in a MATLAB environment based on the surveillance system’s actual test data. In this work, we evaluate the proposed cleaning strategy’s efficiency in terms of energy and time consumption. This paper is organized as follows: Section 2 introduces the design of a Multi-robot cleaning system on WASP robot. Section 3 presents the software system architecture of WASP and its multi-robot design to handle multiple tasks. Section 4 discusses the Human safe distancing surveillance module on the robot. Section 5 discusses the adaptive cleaning method in synergy with the robot’s surveillance system. Section 6 validates the experiments and results conducted under different map scenarios. Finally, Section 7 concludes this study and discusses future research directions. This paper concentrates on the development of a safe social distancing surveillance module on the platform to generate an adaptive velocity behavior model for a multi-robot cleaning system.

## 2. Design of Multi-Purpose Service System

**Figure 1:**
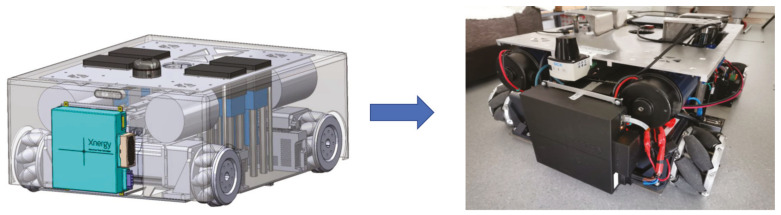
Design of WASP robot.

In this paper, we demonstrate the concept of multi-robot cleaning system on the Intra-reconfigurable logistic platform WASP, as seen in Figure 1. The notion of the WASP platform is to provide:

- Logistics transportation of different kinds of trolleys that are available in hospital settings.
- Safe distancing surveillance system to monitor the human interactions inside hospital environment to control the spread of the virus.
- Efficient cleaning and disinfection in the hospital environment.

The intra-reconfigurability of the robot aims to handle logistics carrying operations and safe distancing surveillance in hospitals, wherein the robot is capable of switching between trolley lifting, trolley towing, and human safe distance monitoring applications. The multi-robot system acts as a virtual extension to the WASP robot to handle cleaning tasks based on the inputs from the human safe distance surveillance system. One of the significant advantages of the WASP design is its intra-reconfigurability along with the multi-robot system, which unifies the model of the robot. Therefore, instead of having different communication frameworks and repetitive use of the same hardware units on different robots’ models, this design requires fewer resources.

### 2.1. Kinematic Motion Model

**Figure 2:**
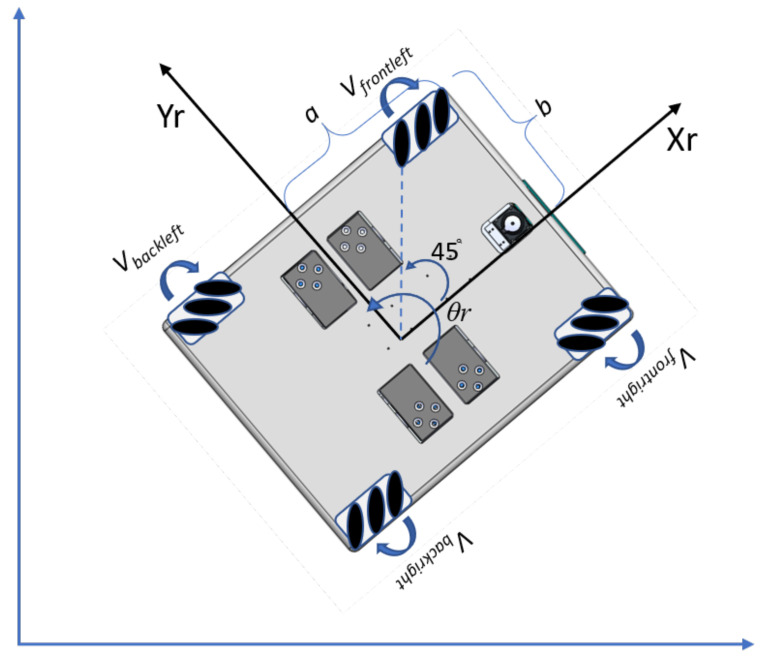
WASP: Kinematic motion model.

The locomotion unit of WASP robot is a four-wheeled mechanism drive system where a series of free moving rollers are attached about the perimeter of the wheels with an angle of 45 degrees. This meccanum wheel design enables the robot to perform motion in $X_{r}$ and $Y_{r}$ direction and $\theta_{r}$ rotation. With the meccanum wheeled drive mechanism, the robot can move longitudinally and laterally, pivot around, and move towards arbitrary direction without changing the orientation. This adds up more degrees of freedom for robot locomotion and can perform logistics applications with smooth navigation even in narrow spaces. For the multi-robot cleaning system, we consider the same mechanical features as the WASP robot. This enables the cleaning system to achieve area coverage in zig-zag-based path planning. Figure 2 describes the wheel configuration and posture definition of the robot where the configuration parameters $V_{frontleft},V_{frontright},V_{backright},V_{backleft}$ specify the corresponding velocity of the wheel of the robot. Parameters *a* and *b* are the distance from the robot’s center in the corresponding *x* and y−axis of the robot frame. Based on the research work done by Taheri et al., in Reference [46], the forward kinematic Equations (1)–(4) and inverse kinematic Equations (5)–(7) of the robot can be written as:

(1) $$\omega_{frontleft}=\frac{1}{r}\left(vel_{y}-vel_{x}+\left(a+b\right)\omega_{\theta }\right),$$

(2) $$\omega_{frontright}=\frac{1}{r}\left(-vel_{y}+vel_{x}+\left(a+b\right)\omega_{\theta }\right),$$

(3) $$\omega_{backright}=\frac{1}{r}\left(vel_{y}+vel_{x}+\left(a+b\right)\omega_{\theta }\right),$$

(4) $$\omega_{backleft}=\frac{1}{r}\left(-vel_{y}-vel_{x}+\left(a+b\right)\omega_{\theta }\right),$$

where $\omega_{frontleft},\omega_{frontright},\omega_{backright},\omega_{backleft}$ represent the corresponding angular velocities of each wheel calculated based on the command velocities $vel_{y},vel_{x},\omega_{\theta }$ received by the robot, and *r* is the radius of each wheel.

(5) $$V_{x}=0.25*\left(V_{frontleft}-V_{frontright}+V_{backright}-V_{backleft}\right),$$

(6) $$V_{y}=0.25*\left(-V_{frontleft}+V_{frontright}+V_{backright}-V_{backleft}\right),$$

(7) $$V_{\theta }=0.25/\left(a+b\right)*\left(V_{frontleft}+V_{frontright}+V_{backright}+V_{backleft}\right).$$

After calculating the velocity of the robot based on inverse kinematics equations, Equations (8)–(10) are used to estimate the position of the robot relative to the robot environment.

(8) $$\dot{x}=\left(V_{x}*\cos(-\dot{\theta }\right)-V_{y}*\sin\left(-\dot{\theta }\right))*\delta_{t},$$

(9) $$\dot{y}=\left(V_{x}*\sin\left(-\dot{\theta }\right)-V_{y}*\cos\left(-\dot{\theta }\right)\right)*\delta_{t},$$

(10) $$\dot{\theta }=V_{\theta }*\delta_{t}.$$

The estimated position and velocity of the robot are published in ROS topic as wheel odometry messages.

### 2.2. Multi-Robot Design

**Figure 3:**
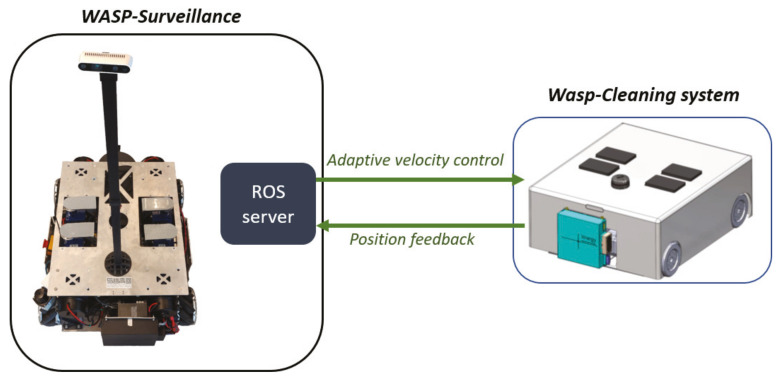
WASP with cleaning subsystem.

In this paper, we propose the design idea of using the multi-robot system to handle cleaning and disinfection tasks. In contrast with the fixed cleaning strategies in the works proposed on the multi-robot cleaning systems [31,33,47,48], this work presents the design idea of using a multi-robot cleaning system to perform adaptive cleaning and disinfection of the environment based on the control commands from the main robot system, as shown in Figure 3. For the ease of demonstration and proof of concept, we use a single sub cleaning robot system to validate the concept of adaptive cleaning method in this paper. For the cleaning system, we replicate the WASP robot’s mechanical design with extra internal space to accommodate for cleaning modules on the robot. On the software side, Robot Operating System (ROS) is used as the underlying software framework to enable the data transmission and processing within the robot system to carry out the designated tasks autonomously. The onboard Nvidia jetson computer is 8 core GPU processor and has 8GB RAM that enables high-level processing.

In this approach, an adaptive cleaning strategy is developed to improve the cleaning and disinfection performance by maximizing area coverage, able to identify contaminated regions in the environment, and clean the environment by spending more time and increasing the power of cleaning modules on the contaminated regions. The control commands for the cleaning system are generated by the WASP platform after processing the heat map generated from the surveillance module. The control model for the adaptive cleaning is generated by adapting to zig-zag-based coverage path planning for maximum area coverage. An adaptive velocity-based behavior model is integrated along with the path generation to enhance the cleaning performance and efficiency by reducing cleaning time and energy consumption. The WASP robot’s ROS framework establishes a virtual wireless communication with the cleaning subsystems over the ROS server through wifi. The control signals generated from the WASP platform are published in a ROS node and exported to the cleaning subsystem to perform the tasks through ROS client-server. The cleaning system sends the position feedback to the WASP robot’s surveillance system to update the position on the map.

## 3. Software System Architecture

**Figure 4:**
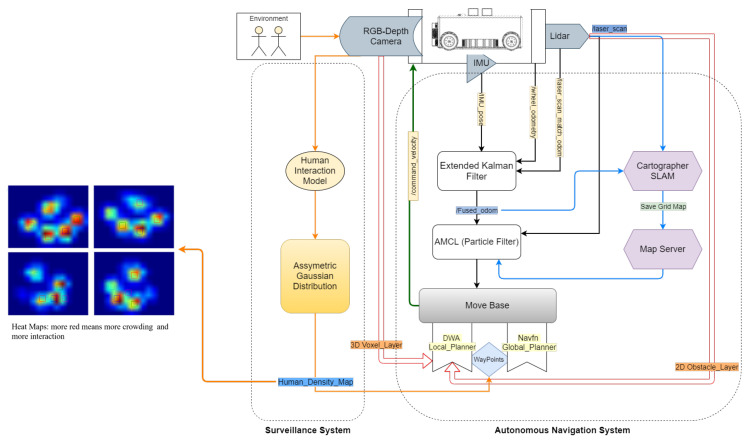
System architecture.

WASP is an Intra-reconfigurable robot designed to handle logistics, safe distance surveillance, and cleaning applications. The platform’s software system is categorized into autonomous navigation, surveillance system, and multi-robot cleaning system, as shown in Figure 4.

For the autonomous navigation of the platform, the robot is equipped with a 2D laser scan range sensor, RGB-depth camera, and an Industrial grade IMU sensor. Robot localization is one of the critical things required for stable and effective autonomous navigation where the pose of the robot is estimated by the sensor fusion from wheel odometry and IMU data through an extended Kalman filter. For dynamic and static obstacle avoidance, the robot uses both local and global planners. While navigating, the robot generates a global path to reach the goal position, and a local path is generated to avoid dynamic obstacles in the environment by aligning towards the global path. For the safe distancing surveillance system, an intel realsense RGB-depth camera is manually connected on top of the WASP robot with the camera fixed at the height of 150 cm above the ground level. The camera has a field of view of 120 degrees and is used for human detection and tracking to monitor safe distancing. Based on the pose estimates of detected humans and the overlapping of each human’s personal space zones, human interaction levels are measured and plotted as a heat map on the grid map.

### Multi-Robot Cleaning System

**Figure 5:**
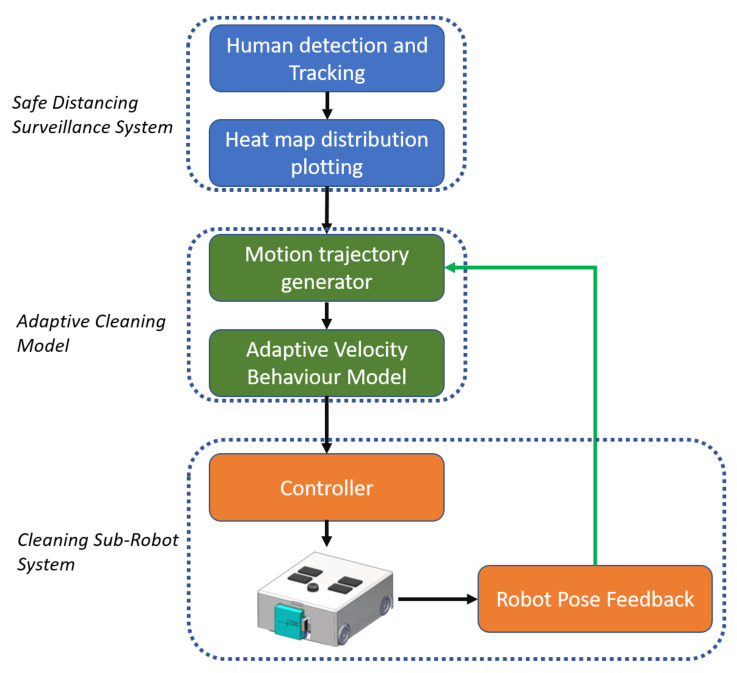
Motion planner framework for sub-robot cleaning system.

For the case of a multi-robot cleaning system, the heat map generated from the WASP robot’s surveillance module is processed to generate a motion trajectory. Figure 5 summarizes the Motion planner framework for the multi-robot cleaning system. The motion trajectory generated for the cleaning system is based on the zig-zag area coverage path planning. Along with motion trajectory generation, velocity control is also generated based on an adaptive velocity behavior model. The velocity for the path is modeled based on the generated heat map of the environment, where the velocity will be minimum at the regions marked with high intensity on the heat map and maximum at regions marked with low intensities. This approach ensures that the cleaning system spends more time cleaning the contaminated areas based on the human interaction levels. The generated control commands are sent to the cleaning robot through the ROS server over wifi, and, in return, the pose feedback of the cleaning robot is sent back to the WASP platform to update the next set of motion control commands.

## 4. Human Safe Distancing Surveillance Framework

**Figure 6:**
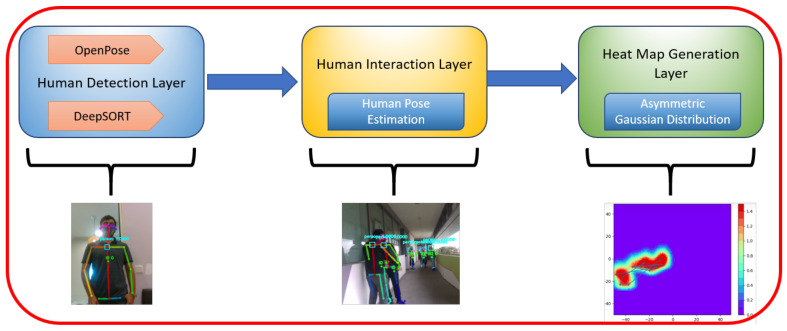
The block diagram of the vision-based framework for safe social distance surveillance.

The objective of the proposed framework for human detection and monitoring human interaction levels is to ensure safe social distancing norms in public places to control the spread of the virus during pandemic situations, especially with the ongoing COVID-19. As the WASP robot has the intra-reconfigurable capability of lifting and towing the trolleys for logistics applications and handle safe social distancing surveillance in large spaces, like hospital settings, good and high-quality perception and navigation sensors are required. Therefore, the robot is mounted with a RealSense D435 RGB-depth sensor as the perception unit and Sick 2D lidars to collect the data from surroundings. In this work, we present a vision-based perceptive framework for safe social distancing surveillance, as shown in Figure 6. The first layer of the perceptive algorithm processes the image stream from an RGB-depth camera and uses deep learning convolutional neural network to handle the human detection tasks. The second layer further processes the detected human data along with human position and orientation to estimate the interaction levels between them. The third layer of this framework is to generate a heat map on a grid map by estimating the cost accumulated in each grid cell based on detected humans and their interaction levels. The detailed description of each layer of the perception framework is explained in the following subsections.

### 4.1. Human Detection Layer

As surveillance is one of the keys that focuses on the software module used on this robotic platform, we use visual feedback from an RGB-Depth sensor to detect and estimate the human’s pose and position in the environment. The vision sensor publishes raw color image topics and depth information through the ROS node. We further extend our software framework with state-of-the-art external perception library OpenPose [49] as an underlying layer on our system to carry out human detection and pose estimation tasks. OpenPose is a framework used to accurately estimate human poses and realize human tracking in real-time applications. It was developed using the VGG pre-trained network model and using Caffe architecture. The pre-trained Openpose model can detect 25 joints for a human body, which is trained with COCO and MPII datasets to extract body keypoint coordinates. Using Openpose ROS wrapper, we create a ROS node to subscribe to the raw image and depth feedback topics published by the RGB-d camera node. The pre-trained Caffe models are run in the background and matched against the camera’s feedback to detect and estimate multiple humans with their poses and determine human key nodes of the detected body parts in the image frame. Each image frame is processed here independently and therefore loses track of previously detected humans. So, on top of this, we implement DeepSort [50] to track detected humans in the environment. For each detection, tracking information of the human is generated along with the image frames. If the detected humans disappear over the later frames, the older humans’ tracking information is eliminated to avoid duplication after applying a threshold gap. Further, a deep learning-based metric approach was used to quantify the association with the Kalman states. In addition, the standard Hungarian algorithm was utilized to associate the data efficiently. To track the human motion, the joint coordinates of the detected human candidate are passed to deepSORT, and an id for each detected human is assigned to perform motion tracking.

### 4.2. Human Interaction Layer

To measure interaction levels between humans, estimation of human attributes, like shape and size, along with their position and orientation in the environment is crucial. Oppose library gives 25 joints positions of the body in 3 dimension array. The first two columns are the X and Y joint positions, while the third one is detection accuracy. We further filter out the false detections based on the threshold accuracy for each detected joint. In this work, we use the detected human’s joint positions to measure the geometrical attributes of a human. For example, the detected human width can be calculated by measuring the distance between right and left shoulders from the skeleton joint array. Another essential information required to measure the human interaction levels is to know the detected humans’ position and orientation. The raw image frame from the RGB-d camera node, which is used as input for Openpose, provides 2d image frames of detected humans with coordinates of the detected humans. However, the coordinates (u,v) estimated are concerning the pixel coordinates and changes within the space of the image frame. So, the pixel coordinates from the raw color image frame further need to be transformed to coordinates in the world frame for estimating human positions in the map of the robot environment.

#### 4.2.1. Transformation from Image to World Frame

The estimated pixel coordinates of detected key points of the person candidate are transformed to World coordinate system using 3D depth information through backward projection. When the robot navigates through the environment, the robot pose is estimated by the particle filter algorithm to keep track of the transform from the global world frame to the base link robot frame. The RGB-D camera used on the robot gives both depth image information and RGB color information. The depth cloud provided by the camera is nothing but an array of depth points, whereas the RGB color image comprises 3 channels in each pixel of the image frame. We further transform the estimated 2D pixel coordinates to a world frame using 3D depth data. The RGB color image frame is given as input to the Openpose trained model for human detection on the image frame. The u and v pixel coordinates of the detected human are extracted from the image frame and are taken from the pixel position of the detected region on the image frame. The depth information provided by the RGB-D camera provides an accurate depth estimate of the detected human from the camera frame. To measure each detected human’s depth distance, an area of 10 × 10 from the center position of the neck is cropped, and the average of the depth from each of the corresponding pixels of the cropped region is calculated. This average depth distance $d_{o}$ is calculated from the Equation (11).

(11) $$d=\frac{\sum_{i\in \Omega }d_{i}}{\Omega },$$

where Ω represents the total number of pixels in a predefined region, and $d_{i}$ is the depth of each pixel. With u,v pixel coordinates of the RGB color image and depth data from the depth image, we perform a transformation operation to estimate the detected human’s position to the robot base frame. Calibration steps are done where the first step is to calculate the extrinsic and intrinsic calibration matrix. The extrinsic calibration matrix specifies the transformation from world to camera coordinates, and intrinsic calibration parameters transform 3D image position to pixel coordinates. These parameters are calibrated using the checkerboard method. The Realsense RGB-depth camera’s intrinsic parameters estimate the focal length, $f_{x}$, and $f_{y}$, and the optical center, $c_{x}$, and $c_{y}$ in intrinsic camera parameters *I*. The extrinsic parameters of the RealSense camera refer to the estimation of orientation that includes translation vector *K* and a rotation vector *Q* of the camera with respect to world coordinates. The extrinsic matrix *S* can be derived from S=[QK]. With the u and v pixel coordinates *p* and the depth value *z*, we can convert the pixel coordinates from the image plane to real-world coordinates *W* using the Equation (12).

(12) p=I∗[QK]∗P=I∗S∗P.

where P,p are world coordinate and pixel coordinate, respectively. Further, by using the ROS of transformation package [51], the transformation from the camera frame is then translated to the base link, the reference frame for the center of the robot. The frame lookup transform creates a reference frame for detected humans and links their position coordinates to the map frame.

#### 4.2.2. Estimating Orientation Angle of the Detected Humans

We calculate the orientation angle of the human by incorporating the depth information *z* and *x* coordinate positions of both the right and left shoulders of the detected human. Using the Equation (13) below, we estimate the angle in the clockwise direction of the human from the detected right and left shoulder joints’ positions. Note that the $\left(x_{r}^{s},z_{r}^{s}\right)$, $\left(x_{l}^{s},z_{l}^{s}\right)$ are the coordinates in (x,z) plane of right shoulder joint and left shoulder of detected human, respectively.

(13) $$angle=atan2(|z_{r}^{s}-z_{l}^{s}|,|x_{r}^{s}-x_{l}^{s}|)\times 180/\pi .$$

However, the orientation angle estimated from the detected shoulder position gives only the rotation angle in an interval of 180 degrees and not the human heading angle. To estimate the human heading angle, we use the detection of key points on the human face to measure the heading angle. If in a case of failure to detect a joint, the OpenPose library will automatically fill a value ’0’ this joint among 25 joints. This behavior is to detect the direction because if the person is heading in the same direction as the camera, it will fail to recognize the human face features, and Thus, there will not be any joint detected on the face. Taking this as a reference for heading direction, the orientation angle estimated can be +180 or +0. Thus, the equations can be modified as (14), in which 0 degree means facing opposite with the camera.

If joint-nose exist:

(14) $$angle=\left(atan2(\right|z_{r}^{s}-z_{l}^{s}\left|,\right|x_{r}^{s}-x_{l}^{s}|)\times 180/\pi )+180,$$

else: angle can find by Equation (13).

### 4.3. Heat Map Generation

The last step of this framework is to generate a heat map distribution based on the personal human space and their interactions with other people. One important aspect of estimating human interaction levels is individual human personal space. In this work, we are particularly interested in the personal space zone that people maintain around themselves. Numerous researches have shown how the shape and size of personal space changes based on human activities, like walking, sitting, and engaging in face-to-face interactions [52,53]. The approximate shape of personal space varies from one person to another, depending on their geometrical attributes. With the idea of personal space, we approximate a cost function based on asymmetrical Gaussian distribution defined in Reference [54] for personal human space to account for their interactions and their geometrical attributes. Equation (15) refers to the asymmetric Gaussian distribution function used to generate a heat map of personal space in considering the points in (x,z) plane.

(15) $$f\left(x,z\right)=exp^{-(a{\left(x-z_{c}\right)}^{2}+2b\left(x-x_{c}\right)\left(z-z_{c}\right)+c{\left(z-z_{c}\right)}^{2})};$$

the shape of human personal space is formed by changing the variances of distribution in 3 standard deviaion directions $\sigma_{h}$, $\sigma_{r}$, and $\sigma_{s}$ frontal, rear, and side spaces, respectively, along with the human heading angle in a Gaussian distribution function. The mean of the distribution is estimated based on the centre coordinate of the detected human in the environment. The algorithm 1 below explains the implementation of asymmetric Gaussian distribution. The coefficients a,b,c in Equation (15) can be derived by the Algorithm 1.



**Algorithm 1: Asymmetric Gaussian-based-heat map generation**

$1:\mathrm{find}:\alpha \leftarrow atan2\left(z-z_{c},x-x_{c}\right)-\theta +\pi /2$

2: Normalize(α)

$3:\;a\leftarrow {\left(cos\theta \right)}^{2}/\left(2\sigma^{2}\right)+{\left(sin\theta \right)}^{2}/\left(2\sigma_{s}^{2}\right)$

$4:\;b\leftarrow sin\left(2\theta \right)/\left(4\sigma^{2}\right)-sin{\left(2\theta \right)}^{2}/\left(4\sigma_{s}^{2}\right)$

$5:\;c\leftarrow {\left(sin\theta \right)}^{2}/\left(2\sigma^{2}\right)+{\left(cos\theta \right)}^{2}/\left(2\sigma_{s}^{2}\right)$

$6:\mathrm{return}(exp\left(-\left(a{\left(x-x_{c}\right)}^{2}+2b\left(x-x_{c}\right)\left(z-z_{c}\right)+c{\left(z-z_{c}\right)}^{2}\right)\right)$



#### Plotting in Grid Maps

The occupancy grid map of the environment is discretized into grid cells with each cell of size 0.05 × 0.05 m. Each cell is assumed to have values ranging from 0 to 1 based on the Asymmetric Gaussian distribution model for human density and interaction levels. The map in the figure shows the environment’s structure where every black pixel shows the space occupied by obstacles, and the white pixel represents the free space in the environment. The other colors in the grid cells show the human-occupied space with the intensity of the distribution ranging from 0 to 1. The maximum peak value of the distribution is represented with the red pixel, and the minimum value in the distribution is represented with the blue pixel on the grid-based heat map. So, the distribution intensity is represented as a variation in the heat map color. For the case of multiple people standing closer, the distribution values will be accumulated over the grid space and are normalized. So, the regions that are overlapped by the spatial zones of multiple persons will have a peak value of the grid cells, representing an area of highly contaminated space. The tracking id of the detected human is stored in the memory. While navigating, if the robot detects a person, an id will be created for the person, and if the same id is detected again on the same position of the grid space, the heat distribution is not generated. The new distribution is generated if the new id appears or the tracked id moves to different grid cells. The generated distributions are accumulated on the grid-based heat map. The heat map density on the distribution is estimated based on the level of interactions between humans.

## 5. Adaptive Velocity Model

The WASP platform, being the brain/master unit of this system, processes the heat map generated from the surveillance module to generate an adaptive velocity behavior model based on a zig-zag path planner for the cleaning system. The heat map distribution result is used as input to the velocity behavior model for disinfection robots to efficiently clean the areas having peak values on the generated heat map. The robot calculates the velocity with which the robot should traverse to sterilize the environment efficiently. The higher the heat map’s value, the slower it to disinfect the region. On the other hand, the conventional cleaning systems use a uniform velocity model; thus, there are chances of leaving the regions uncleaned. So, this work demonstrates the implementation of an adaptive velocity behavior model based on both linear and non-linear functions to evaluate the performance of cleaning in terms of energy and time consumption with respect to the heat map distribution values.

### 5.1. Linear Function

To model linear adaptive velocity behavior function, we use parameters, such as maximum velocity and minimum velocity, required for the cleaning robot to clean and the probability of the contaminated grid cell. For this approach, we considered maximum velocity to clean the environment as 0.2 m/s and minimum velocity to clean the peak contaminated regions as 0.05 m/s and modeled a linear equation, as shown in Equation (16).

(16) $$v_{cell}=\left(-0.15*P\left(z_{cell}\right)+0.2\right),$$

where $v_{cell}$ is the velocity of the robot in the particular cell, and $P(z_{cell})$ is the probability of the cell region being contaminated.

### 5.2. Non-Linear Function

Similar to the linear function approach, we model the non-linear adaptive velocity behavior model by considering a maximum velocity of 0.2 m/s and minimum velocity greater than 0 m/s to clean the environment. We considered two non-linear functions based on tanh and sigmoid function to multiply with the linear function in Equation (16) to add non-linearity to the velocity behavior model. Equation (17) is the non-linear adaptive velocity behavior model based on tanh function with maximum velocity of 0.2 m/s and minimum velocities of 0.0119 m/s. Equation (18) is based on the sigmoid function with maximum velocity of 0.2 m/s and minimum velocities of 0.0268 m/s.

(17) $$v_{cell}=\left(-0.15*P\left(z_{cell}\right)+0.2\right))*\left(1-tanh\left(z_{cell}\right)\right),$$

(18) $$v_{cell}=\frac{1}{1+e^{z_{cell}}}*2\left(-0.15*P\left(z_{cell}\right)+0.2\right)).$$

Based on the velocity models, we further implement zig-zag-based path planning strategy to perform selective cleaning over the contaminated regions by inducing more cleaning power and low velocities, unlike the other conventional cleaning robots. We evaluate the proposed adaptive velocity behavior model and path planning strategy with respect to time consumption and energy conserved.

## 6. Energy Consumption Estimation Model

**Figure 7:**
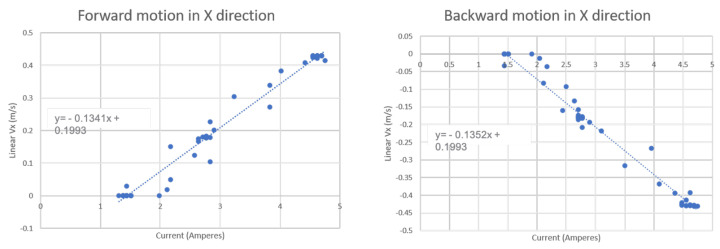
Relation between Current consumption and Linear Vx for the cleaning robot.

**Figure 8:**
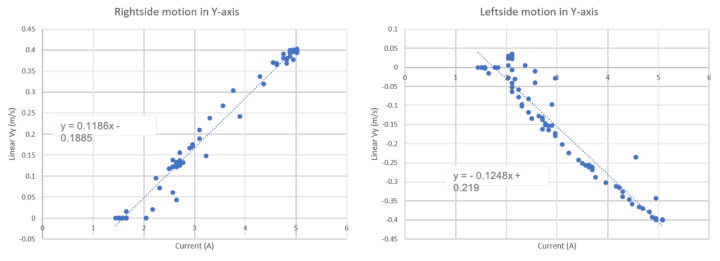
Relation between Current consumption and Linear Vy for the cleaning robot.

The energy consumption during the area coverage for the virtually connected floor cleaning system is directly related to the navigation trajectory and adaptive velocity behavior model generated by the WASP platform. To calculate the total energy consumed based on the motion control function generated by the adaptive velocity behavior model, we measure the energy consumption for a series of robot actions encountered during cleaning. We use the battery management system on the robot to record the current reading of the robot for each action performed by the robot. Figure 7 shows the robot’s current readings for the robot in forward and backward motions in the X−axis with their respective linear velocities Vx. Similarly, Figure 8 displays the current readings of the robot for left and right directional motions in the Y−axis with linear velocities Vy. We approximate the relationship between the current readings and the robot velocities using linear functions as in (20)–(21) where *I* is the current consumed.

(19) $$I=\frac{V_{x}}{0.1341}+1.441\;\left(Forward\;motion\right),$$

(20) $$I=\frac{-V_{x}}{0.1352}+1.441\;\left(Backward\;motion\right),$$

(21) $$I=\frac{V_{y}}{0.1186}+1.58\;\left(Rightward\;motion\right),$$

(22) $$I=-0.1248\frac{-V_{y}}{0.1248}+1.754\;\left(Leftward\;motion\right).$$

This linearly approximated energy vs velocity equation is used to estimate the total energy consumption for the proposed adaptive velocity behavior model.

Table 1 summarizes the energy consumption for each robot’s actions with corresponding velocities. The measured energy values for the corresponding velocity of the robots are approximated by a linear distribution. Further, for the cleaning module’s energy consumption on the robot, we considered the use of vacuum powered suction unit rated at 12 V and 5 A with 6500 Pa pressure.

Based on each robot action’s energy consumption, we estimate the robot’s total energy consumption in the experiments and results section for each function model based on adaptive velocity behavior functions.

## 7. Experiment, Results and Discussion

### 7.1. Experimentation

In this work, the algorithms implemented to solve the performance issues of area coverage path planning on the minimization of time and power consumption of cleaning robots are validated. As part of this research work, we evaluated the WASP cleaning robot’s performance in MATLAB simulation and validated the proposed adaptive velocity behavior model for cleaning by organizing experiments under four different scenarios. We used different map environments for each scenario and conducted experiments by gradually increasing human density and interaction levels for each scenario. The total grid space for each map environment is defined with a resolution of 0.05 m. The generated occupancy grid map is decomposed into a mesh-grid with each cell of size 0.05 m. After performing human detection and estimating interaction levels, the heat map is generated, and the values are plotted on the map’s grid cells. The values accumulated on each grid cell range from 0 to 1, depending on the human interaction levels. With the generated heat map distribution on the grid cells, the robot produces an adaptive velocity behavior model to navigate and perform area coverage for scenarios I to IV. We compare the adaptive velocity behavior model results using the linear function and non-linear functions (tanh and sigmoid) with a uniform velocity behavior model similar to conventional cleaning robots. We validated the experimental results by simulating the Adaptive velocity behavior model with time consumption and energy consumption on the WASP cleaning platform. For scenarios I-IV, the experiments were run on a predefined workspace with dimensions and number of persons as listed in the Table 2.

**Figure 9:**
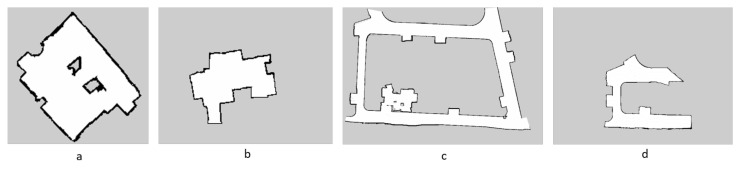
Generated occupancy grid maps of the work space environments for scenarios I to IV.

Figure 9a–d represents the maps of the environments for scenarios I to IV.

### 7.2. Results and Analysis

**Figure 10:**
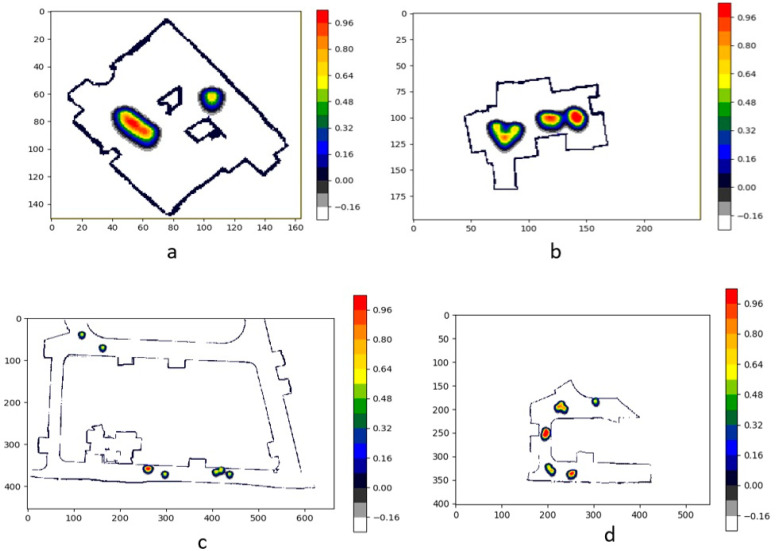
Heat map distribution plotted on the grid maps for scenarios I to IV based on the human interactions.

The experimental trials were initiated by running the robot in the testbed environment to monitor the human interactions and safe distancing. The robot generates a heat map concerning the camera frame and records the detected humans’ pose. This data is then processed in Python to perform the transformation from camera frame to map frame and generate heat map plotting on grid map, as shown in Figure 10a–d.

**Figure 11:**
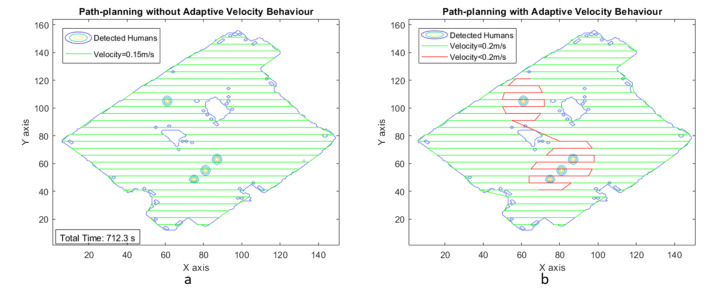
Zig-zag-based area coverage path generated for scenario I.

The second phase of experimental trials was simulated in MATLAB software to generate an adaptive velocity behavior model for a zig-zag-based area coverage approach. Figure 11a illustrates the area coverage path planning generated for scenario I with uniform velocity model, and Figure 11b illustrates the area coverage path planning for scenario I with Adaptive velocity behavior model. The path with green color indicates the regions where the robot navigates with maximum velocity. The red color path indicates the regions with lower velocities to spend more time cleaning the contaminated regions.

**Figure 12:**
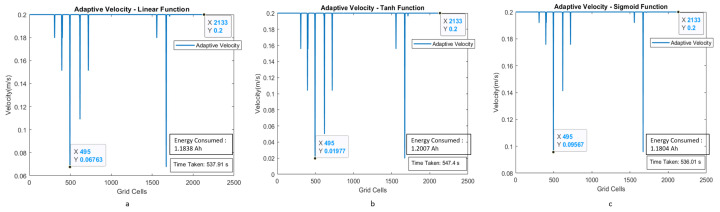
Adaptive velocity behavior model with linear, tanh, and sigmoid functions with velocity corresponding to the grid cells of the map for scenario I.

The estimated time taken to cover the region with a uniform velocity model with a velocity of 0.15 m/s is around 712.3 s. The energy consumption for the entire area coverage process for the conventional cleaning method is 1.4958 Ah of the total battery capacity of 36 Ah. Figure 12a–c shows the plot between velocity and grid cell position on the map for the case of adaptive velocity behavior model with linear, tanh, and sigmoid functions. The results validate that time taken to cover the area for the scenario I is less for the adaptive velocity behavior model based on the sigmoid function, and the energy consumption is almost similar for all three adaptive velocity functions. The lowest energy consumption reading is recorded for the sigmoid-based behavior model with 1.1804 Ah, which is 3.27 percent of the total battery capacity.

**Figure 13:**
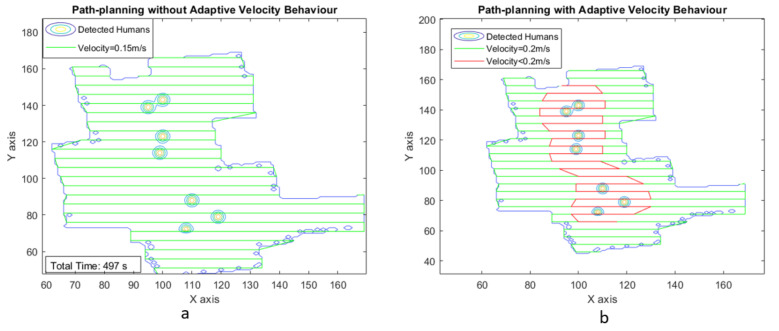
Zig-zag-based area coverage path generated for scenario II.

Similarly, for scenario II, we increased the number of humans interacting inside the testbed environment. After completely monitoring the human interactions in the defined space using the surveillance module, the generated heat map is processed to generate an area coverage path plan for both uniform and adaptive velocity models, as shown in Figure 13

**Figure 14:**
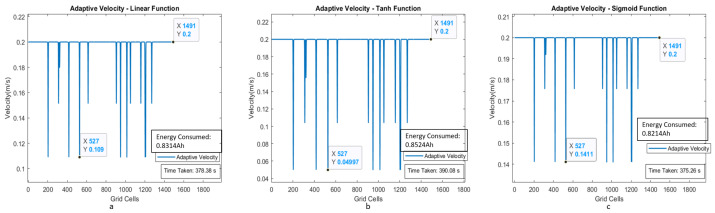
Adaptive velocity behavior model with linear, tanh, and sigmoid functions with a velocity corresponding to the map’s grid cells for scenario II.

With uniform velocity model, the estimated time for area coverage is 497 s and the estimated energy consumption for the cleaning task is 1.0436 Ah, which is 2.89 percentage of the total battery. Figure 14a–c illustrates the plot between velocity and grid cell position on the map for the case of adaptive velocity behavior model with linear, tanh, and sigmoid functions.

**Figure 15:**
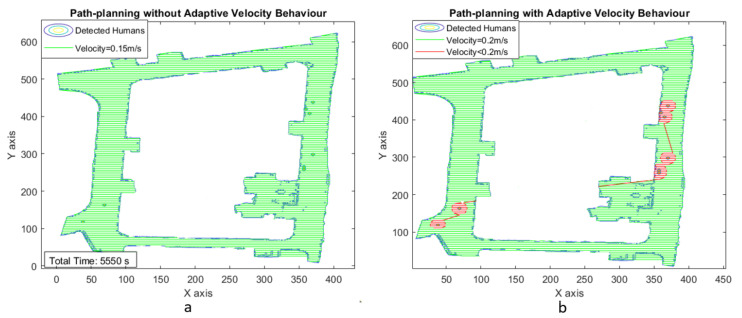
Zig-zag-based area coverage path generated for scenaio III.

**Figure 16:**
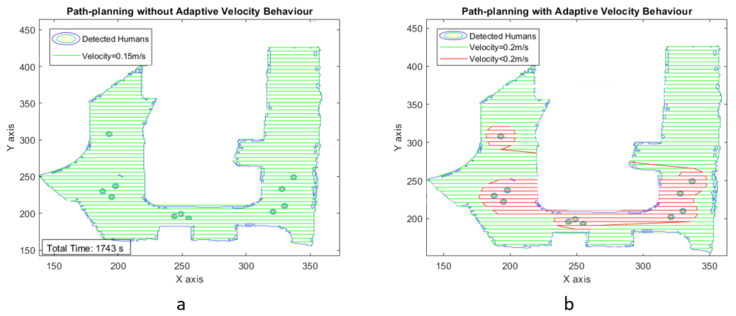
Zig-zag-based area coverage path generated for scenario IV.

With the experiments in scenarios III and IV, we considered a larger testbed of dimensions 27 × 20 and 13.5 × 9 m, respectively. Moreover, the increased number of humans in the space (8 in scenario III and 11 in scenario IV) to validate the efficiency of the proposed adaptive velocity behavior model. Figure 15 and Figure 16 show the area coverage path plan for both uniform and adaptive velocity behavior models for scenarios III and IV.

**Figure 17:**
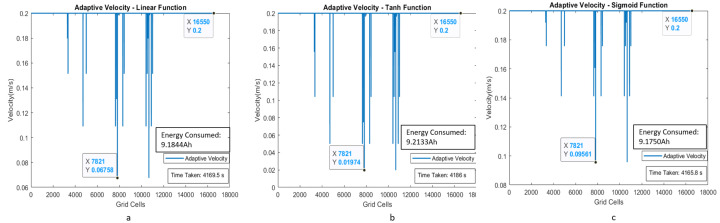
Adaptive velocity behavior model with linear, tanh, and sigmoid functions with velocity corresponding to the grid cells of the map for scenario III.

**Figure 18:**
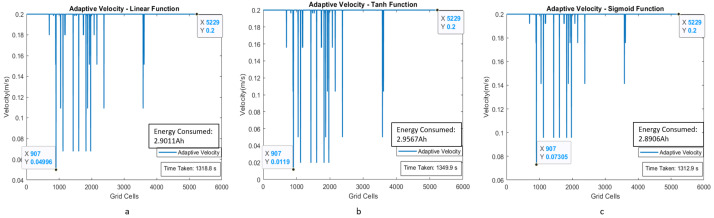
Adaptive velocity behavior model with linear, tanh, and sigmoid functions with a velocity corresponding to the grid cells of the map for scenario IV.

Figure 17 and Figure 18 illustrate the adaptive velocity model plot with grid cell position for scenarios III and IV. Even though human interaction levels and map dimensions are increased, the robot can still achieve area coverage with less time frame and less energy consumption while using the adaptive velocity behavior model. The experimental results are summarized in Table 3 and Table 4 for time and energy, respectively, which indicates that the robot could perform cleaning faster using the adaptive velocity model, especially with sigmoid function.

## 8. Conclusions

This paper presents a novel multi-robot-based adaptive cleaning model in synergy with the human interaction heat map generated by a human safe distancing surveillance system on WASP intra-reconfigurable robot. This cleaning methodology is demonstrated to sterilize large space environments, like hospitals, shopping malls, or high-rise construction buildings, in response to the global pandemic caused due to the COVID-19. We introduced the mechanism design and software modules of the WASP robot and successfully demonstrated the ability to handle a safe distancing surveillance system along with a multi-robot cleaning method. In addition, we introduced adaptive velocity behavior models based on linear and non-linear functions to compare the cleaning performance with the uniform velocity model used in conventional cleaning robots. Experiments were performed in four different map environments by gradually increasing map size and human density to evaluate the performance of the proposed zig-zag path-based adaptive cleaning approach in terms of time and energy consumption. In all the experimental scenarios, the adaptive velocity behavior model based on the non-linear sigmoid function has shown superior cleaning performance in energy and time consumption. Moreover, the results using linear function it is similarly to results based on sigmoid function. In future works, we aim to implement the proposed adaptive cleaning approach for different coverage path planning algorithms and demonstrate the real-time cleaning performance on multi-robot cleaning systems.

## Figures and Tables

**Table 1:** Energy consumption.

	Voltage Rate(V)	Rated Current(A)
Forward Motion	48	$\frac{V_{x}}{0.1341}+1.441$
Backward Motion	48	$\frac{-V_{x}}{0.1352}+1.441$
Rightward Motion	48	$\frac{V_{y}}{0.1186}+1.58$
Leftward Motion	48	$-0.1248\frac{V_{y}}{0.1186}+1.754$
Vacuum Unit	12	4.5

**Table 2:** Experimental Testbed.

Scenarios	Dimensions(Metres)	Total Number of Humans in the Testbed
Scenario I	5.3 × 5	4
Scenario II	5.7 × 3.7	7
Scenario III	27 × 20	8
Scenario IV	13.5 × 9	11

**Table 3:** Total estimated time (in seconds) for each scenario under various models.

Scenarios	Uniform Model	Tanh Function	Linear Function	Sigmoid Function
Scenario I	712.3 s	547.4 s (↓23.1%)	537.91 s (↓24.48%)	536.01 s (↓24.74%)
Scenario II	497 s	390.08 s (↓21.51%)	378.38 s (↓23.86%)	375.26 s (↓24.49%)
Scenario III	5550 s	4186 s (↓24.57%)	4169.5 s (↓24.8%)	4165.8 s (↓24.94%)
Scenario IV	1743 s	1349.9 s (↓22.5%)	1318.8 s (↓24.3%)	1312.9 s (↓24.6%)

**Table 4:** Total estimated energy consumption for each scenario under various models (in percentages with total battery capacity of 36 Ah ).

Scenarios	Uniform Model	Tanh Function	Linear Function	Sigmoid Function
Scenario I	4.15	3.33 (↓19.7%)	3.28 (↓20.9%)	3.27 (↓21.2%)
Scenario II	2.89	2.36 (↓18.33%)	2.3 (↓20.4%)	2.28 (↓21.1%)
Scenario III	32.37	25.59 (↓20.9%)	25.51 (↓21.19%)	25.48 (↓21.28%)
Scenario IV	10.16	8.21 (↓19.19%)	8.05 (↓20.76%)	8.02 (↓21%)

## Data Availability

Not applicable.
